# Association of different piperacillin/tazobactam ratios and dosages with the risk of hypokalemia: a propensity score-matched retrospective cohort study

**DOI:** 10.3389/fcimb.2026.1959126

**Published:** 2026-10-01

**Authors:** Yuan-min Wang, Bi-ying Zhao, Cang-hong Ouyang, Fu-kun Zhao, Yang Jiao, Min Luo

**Affiliations:** 1 Clinical Pharmacy Department, The Third Affiliated Hospital of Zunyi Medical University, The First People's Hospital of Zunyi, Zunyi, Guizhou, China; 2 Department of Preventive Healthcare, The Third Affiliated Hospital of Zunyi Medical University, The First People's Hospital of Zunyi, Zunyi, Guizhou, China; 3 Department of Cardiology, The Third Affiliated Hospital of Zunyi Medical University, The First People's Hospital of Zunyi, Zunyi, Guizhou, China

**Keywords:** FAERS database, hypokalemia, penicillin-class antibiotics, piperacillin/tazobactam, propensity score matching

## Abstract

**Background:**

To investigate the association of different piperacillin/tazobactam (TZP) ratios and dosages with hypokalemia, to characterize hypokalemia-related disproportionality signals for selected penicillin-class antibiotics, and to discuss potential mechanistic explanations in relation to chemical structure, with the aim of providing evidence for rational clinical medication use.

**Methods:**

This single-center retrospective cohort study included hospitalized patients who received TZP at the First People’s Hospital of Zunyi between January 1, 2020 and January 25, 2025. TZP ratio and dosage were defined as exposure variables, and hypokalemia (serum potassium <3.5 mmol/L after TZP initiation) was the clinical outcome. Propensity score matching (PSM) was used to balance measured baseline characteristics, laboratory parameters, infection sites, and concomitant medication categories. In addition, reports involving selected penicillin-class antibiotics were retrieved from the U.S. Food and Drug Administration Adverse Event Reporting System (FAERS). Reporting odds ratio (ROR), proportional reporting ratio (PRR), and corresponding 95% confidence intervals (95% CIs) were calculated to identify disproportionality signals; these measures were interpreted as reporting signals rather than incidence, clinical risk, or causality.

**Results:**

A total of 1,825 patients treated with TZP were included. In the ratio comparison, 678 patients received TZP at a ratio of 8:1 and 716 received TZP at a ratio of 4:1. In the dosage comparison, 678 patients received TZP 3.375 g q8h and 431 received TZP 4.5 g q8h. After 1:1 PSM, 1,356 patients were matched in the ratio groups, with 678 patients in each group, and the incidence of hypokalemia did not differ significantly (33.186% vs. 33.481%; *P* = 0.908). In the dosage groups, 862 patients were matched, with 431 patients in each group. The incidence of hypokalemia was 33.643% in the TZP 3.375 g q8h group and 40.139% in the TZP 4.5 g q8h group (*P* = 0.048, χ² = 3.907). In FAERS, TZP (ROR = 3.809, PRR = 3.786, χ² = 71.205) and ampicillin (ROR = 5.326, PRR = 5.287, χ² = 58.414) met the prespecified criteria for positive hypokalemia disproportionality signals.

**Conclusion:**

Within this TZP-treated cohort, the 8:1 and 4:1 component ratios were not associated with a detectable difference in hypokalemia incidence, whereas the 4.5 g q8h regimen was associated with a higher observed incidence than the 3.375 g q8h regimen after matching. FAERS findings support the presence of hypokalemia reporting signals for TZP and ampicillin but do not establish comparative clinical risk or causality. These observations support careful serum potassium monitoring during TZP therapy, particularly with higher-dose regimens.

## Graphical Abstract

**Figure:**
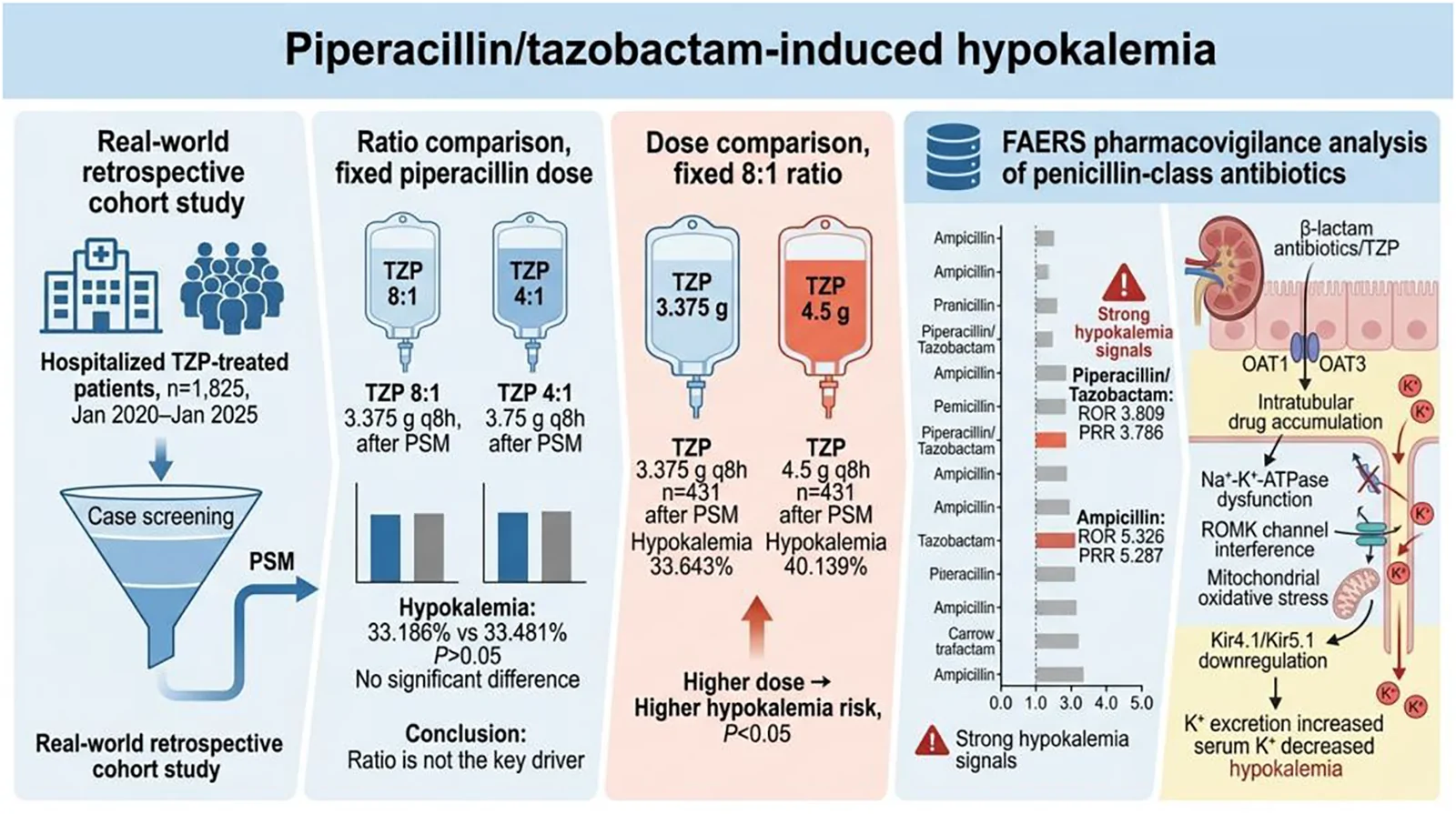
This graphical abstract summarizes the association between piperacillin/tazobactam (TZP) dosing and hypokalemia risk. In a real-world retrospective cohort of 1,825 hospitalized TZP-treated patients, propensity score matching showed no significant difference in hypokalemia between the TZP 8:1 and 4:1 regimens at a fixed piperacillin dose. In contrast, at a fixed 8:1 ratio, the higher-dose regimen was associated with a greater risk of hypokalemia. FAERS analysis identified strong hypokalemia signals for piperacillin/tazobactam and ampicillin. A proposed mechanism involves renal tubular uptake through OAT1/OAT3, intratubular drug accumulation, disruption of potassium transport pathways, increased urinary potassium loss, and reduced serum potassium levels. These findings support serum potassium monitoring during TZP therapy, particularly with higher doses or prolonged exposure.

## 1 Introduction

Piperacillin/tazobactam (TZP) is a fixed-dose combination of piperacillin, a penicillin-class antibacterial agent, and tazobactam, a *β*-lactamase inhibitor. It is widely used for the treatment of moderate-to-severe bacterial infections, such as pneumonia, intra-abdominal infection, and sepsis (Wang et al., 2026). In recent years, electrolyte disturbances associated with TZP, particularly hypokalemia, have drawn increasing clinical attention. In October 2020, the Pharmaceuticals and Medical Devices Agency (PMDA) of Japan issued a safety alert requiring revision of the TZP package insert to include a warning about the risk of hypokalemia, reflecting growing regulatory attention to this safety issue (Center for Drug Reevaluation, 2020). Clinically, hypokalemia may cause muscle weakness, arrhythmia, and even cardiac arrest, and may have a considerable adverse effect on the prognosis of critically ill patients.

Previous studies (Kuramoto et al., 2019; Seo and Kim, 2022; Cao et al., 2023) have reported that the incidence of TZP-associated hypokalemia ranges from 12.6% to 30.1%, with dosage, age, sex, and baseline serum potassium level identified as potential influencing factors. Studies published over the past five years have mainly focused on risk factor analysis. Sun Jian et al. found that elderly and female patients had a higher risk of hypokalemia (Sun et al., 2025). Sun et al. (2024) compared the safety of TZP preparations with different component ratios in patients with severe pneumonia. Wang et al., (2026) reported that female sex, advanced age, and low baseline serum potassium level were risk factors for TZP-induced hypokalemia. Cao et al. (2023), using data from the Chinese Hospital Pharmacovigilance System (CHPS), further confirmed these risks. In international studies, Seo and Kim (2022) reported in 2022 that the daily dose of TZP was positively associated with the occurrence of hypokalemia (OR = 1.10), while Kuramoto H et al. reported in 2019 that the risk increased significantly in elderly patients aged >80.5 years (Kuramoto et al., 2019). Several studies (Wang et al., 2026; KavyaDeepu and Sekar, 2025; Jansen et al., 2024) have suggested that TZP-induced hypokalemia may be related to increased renal tubular potassium excretion; however, the specific effects of dose dependence and component-ratio differences remain unclear.

As a fixed-dose combination, TZP contains both piperacillin and the *β*-lactamase inhibitor tazobactam, but the contribution of the inhibitor component and the association between different TZP dosages and hypokalemia have not been adequately examined. In addition, few studies have explored whether hypokalemia reports for penicillin-class antibiotics differ according to individual agents or whether such patterns may be related to chemical structure. In real-world clinical studies, treatment groups are usually not randomly assigned, and between-group confounding may affect the reliability of observed exposure-outcome associations. Propensity score matching (PSM) is a useful method for balancing measured confounders and has been widely applied in observational comparative-effectiveness studies based on real-world data (Chen et al., 2024; Hu et al., 2024; Reiffel, 2020). Therefore, the objectives of the present study were: (1) to compare the incidence of hypokalemia between TZP 8:1 and 4:1 component-ratio regimens at a fixed piperacillin dose; (2) to compare the incidence of hypokalemia between TZP 3.375 g q8h and 4.5 g q8h regimens at a fixed 8:1 ratio using PSM to balance measured covariates; and (3) to characterize hypokalemia disproportionality signals for selected penicillin-class antibiotics in FAERS and explore plausible structure-related mechanisms. The clinical cohort and FAERS analyses were considered complementary but methodologically distinct, and the FAERS analysis was not intended to estimate incidence or causal risk.

## 2 Materials and methods

### 2.1 Materials

#### 2.1.1 Data sources

This single-center retrospective cohort study used clinical data from the First People’s Hospital of Zunyi and was approved by the Ethics Committee of the First People’s Hospital of Zunyi [Approval No. Ethics (2025)-1-323]. For the pharmacovigilance component, reports involving penicillin-class antibiotics were retrieved from the U.S. Food and Drug Administration FAERS from the first quarter of 2004 to the fourth quarter of 2024.

#### 2.1.2 Study population

Hospitalized patients who initiated TZP at the First People’s Hospital of Zunyi between January 1, 2020 and January 25, 2025 were screened to establish the retrospective cohort. The index date was the first documented TZP administration. Baseline characteristics and the last serum potassium value before TZP initiation were obtained from the available hospital records, and patients were followed through the documented TZP treatment period using subsequent serum potassium measurements to identify the outcome. The inclusion criteria were as follows: ① age ≥18 years, regardless of sex; ② treatment with piperacillin sodium/tazobactam sodium for more than 2 days; ③ at least one serum potassium measurement both before and after piperacillin sodium/tazobactam sodium administration; and ④ the last serum potassium level before piperacillin sodium/tazobactam sodium administration was ≥3.5 mmol/L. The exclusion criteria were as follows: ① incomplete clinical data; ② receipt of peritoneal dialysis or hemodialysis; and ③ documented conditions likely to independently alter serum potassium, including alkalosis, vomiting, diarrhea, tube drainage, hypomagnesemia, hypokalemic thyrotoxic periodic paralysis, primary hyperaldosteronism, Cushing’s syndrome, and renal tubular acidosis. These baseline conditions were required to have been documented prior to the first TZP administration, rather than representing outcomes occurring after drug initiation.

Using the Medical Dictionary for Regulatory Activities (MedDRA) preferred term “Hypokalemia” and the drug terms “Piperacillin tazobactam”, “Amoxicillin clavulanic acid”, “Amoxicillin”, “Ampicillin”, “Oxacillin”, and “Penicillin G”, reports with documented use of any of the six target penicillin-class antibiotics and a hypokalemia adverse-event report were extracted from FAERS as the pharmacovigilance sample. Because FAERS is a spontaneous reporting system, these reports were used for signal detection only and were not used to calculate event incidence.

### 2.2 Methods and procedures

#### 2.2.1 Data extraction and entry

According to the inclusion criteria, hospitalized patients who received TZP at the First People’s Hospital of Zunyi between January 1, 2020 and January 25, 2025 were screened using the hospital Prescription Automatic Screening System (PASS), an electronic medication-order review and clinical data retrieval platform used by the institution. The extracted variables included basic patient information, vital signs, routine blood test results, underlying comorbidities, including hypertension, myocardial infarction, congestive heart failure, peripheral vascular disease, cerebrovascular disease, dementia, chronic obstructive pulmonary disease, renal disease, diabetes mellitus, liver disease, and cancer; concomitant medications, including nonsteroidal antipyretic/analgesic/anti-inflammatory drugs, angiotensin-converting enzyme inhibitors, angiotensin receptor blockers, *β*-receptor blockers, digoxin, potassium-sparing diuretics, potassium-wasting diuretics, insulin, and glucocorticoids; infection sites, including the lung, abdomen, skin and soft tissue, bone, brain, and urogenital system; dosage regimen; duration of TZP use; baseline serum potassium before medication; the lowest serum potassium value after medication; the serum potassium value at the first occurrence of hypokalemia after medication; blood gas analysis results; and other relevant data. Hypokalemia in the clinical cohort was defined as any documented serum potassium concentration <3.5 mmol/L after TZP initiation. Length of hospital stay and potassium-containing intravenous fluid administration were not analyzed as separate variables in the current dataset. The data were screened according to the exclusion criteria and entered into Excel to establish the analysis database.

For penicillin-class antibiotics, reports of hypokalemia associated with the documented use of six target drugs, namely piperacillin/tazobactam (TZP), amoxicillin/clavulanic acid, amoxicillin, ampicillin, oxacillin, and penicillin G, were extracted from the FAERS database. Drug names were standardized to generic names according to the world health organization drug dictionary and the FDA drug information database, and the generic name was used as the unique identifier for statistical analysis. Reports with missing, obviously incorrect, or duplicate information on drug names or adverse event descriptions were excluded. The eligible data were then entered into Excel to establish the database.

#### 2.2.2 Grouping

After fixing the piperacillin dose, patients were divided according to the fixed component ratio of the drug used, namely piperacillin: tazobactam, into an 8:1 ratio group (3.375 g q8h ivgtt) and a 4:1 ratio group (3.75 g q8h ivgtt). After fixing the TZP component ratio at 8:1, patients were divided according to the dose per administration into a 3.375 g q8h group and a 4.5 g q8h group.

#### 2.2.3 Statistical analysis

Data were processed using R software version 4.2.3 and Python software version 3.11.4. Propensity score matching was performed between groups at a 1:1 ratio using the following measured covariates: sex, age, BMI, hypertension, myocardial infarction, congestive heart failure, cerebrovascular disease, peripheral vascular disease, dementia, chronic obstructive pulmonary disease, renal disease, diabetes mellitus, liver disease, cancer, ALB, ALP, AST, ALT, serum creatinine, creatinine clearance, total bilirubin, platelet count, pulmonary infection, abdominal infection, urinary system infection, skin and soft tissue infection, brain infection, bone infection, the last serum potassium value before TZP treatment, use of medications that may increase serum potassium, and use of medications that may decrease serum potassium. Based on the medication classes recorded in the source dataset, the potassium-increasing category comprised angiotensin-converting enzyme inhibitors, angiotensin receptor blockers, *β*-receptor blockers, digoxin, potassium-sparing diuretics, and nonsteroidal anti-inflammatory drugs, whereas the potassium-decreasing category comprised potassium-wasting diuretics, insulin, and glucocorticoids. These heterogeneous classes were combined into binary categories for matching; individual drug dose, timing, duration, and potency were not modeled. Potassium-containing intravenous fluids were not separately captured as a time-varying covariate. The absolute standardized difference (ASD%) was used to assess between-group balance before and after matching, with ASD <10% considered indicative of acceptable balance. PSM can balance measured covariates only and cannot eliminate residual confounding from unmeasured factors such as infection severity, sepsis status, clinician treatment selection, or rapidly changing physiology. Normally distributed continuous variables were expressed as x ± s. Paired t-tests were used for within-group comparisons, and t-tests were used for between-group comparisons. Categorical variables were expressed as n (%), and the chi-square (χ²) test was used. The significance level (α) was set at 0.05.

In spontaneous reporting systems, signal detection for adverse drug events (ADEs) is commonly performed using disproportionality analysis based on a four-cell contingency table. In this study, the reporting odds ratio (ROR) and the Medicines and Healthcare products Regulatory Agency (MHRA) proportional reporting ratio (PRR) criteria were used to identify whether hypokalemia was reported disproportionately for each target antibiotic compared with other reports in the database. Because disproportionality analysis is a rapid, low-cost screening tool capable of handling large volumes of spontaneous reports, ROR and PRR serve as early-warning signals that flag potentially relevant drug-event combinations for priority investigation. They generate hypotheses regarding a possible association between the target antibiotic and hypokalemia that warrant confirmation and clinical interpretation through subsequent, more rigorous studies. The prespecified signal criteria were a lower 95% CI limit for ROR >1 together with the MHRA criteria (PRR ≥2, χ² ≥4, and at least three reports) (Chen et al., 2025; Wang et al., 2026; Ni et al., 2026). Data processing and formula calculations were performed using Microsoft Excel 2021. The relevant contingency table definitions and calculation methods are shown in **Tables 1**, **2**.

**Table 1:** Fourfold table method for disproportionality analysis.

Adverse event	Target drug	Other drug	Total
adverse drug reaction of interest (ADR)	a	b	a + b
other adverse drug reactions	c	d	c + d
Total	a + c	b + d	a + b + c + d

**Table 2:** Formulas related to ROR and MHRA.

Algorithm	Formula	Threshold range
ROR	ROR = (a/c)/(b/d) 95% CI = exp[ln(ROR) ± 1.96√(1/a + 1/b + 1/c + 1/d)]	Lower limit of 95% CI > 1; a ≥ 2
MHRA	PRR = [a/(a+b)]/[c/(c+d)] χ² = (ad−bc)²(a+b+c+d)/[(a+b)(c+d)(a+c)(b+d)]	PRR ≥ 2; χ² ≥ 4; a ≥ 3

a, reports containing both the target drug and hypokalemia; b, reports containing hypokalemia with other drugs; c, reports containing the target drug with other adverse events; d, reports containing other drugs with other adverse events. ROR, reporting odds ratio; PRR, proportional reporting ratio; MHRA, Medicines and Healthcare products Regulatory Agency; CI, confidence interval.

## 3 Results

### 3.1 Case screening and matching process

**Figure 1** presents the case screening and matching process. From January 1, 2020 to January 25, 2025, a total of 1,825 eligible cases were identified at the First People’s Hospital of Zunyi according to the predefined inclusion and exclusion criteria. Among them, 1,394 cases were included in the component-ratio comparison, including 678 cases in the TZP 8:1 ratio group (3.375 g q8h) and 716 cases in the TZP 4:1 ratio group (3.75 g q8h). After 1:1 PSM, 1,356 cases remained for the final analysis, with 678 cases in each group. The post-matching ASD values were generally below 10%, indicating acceptable balance of the measured baseline covariates between the ratio groups; these balance diagnostics should not be interpreted as evidence regarding associations between the covariates and hypokalemia, as shown in **Figure 2A**.

**Figure 1:**
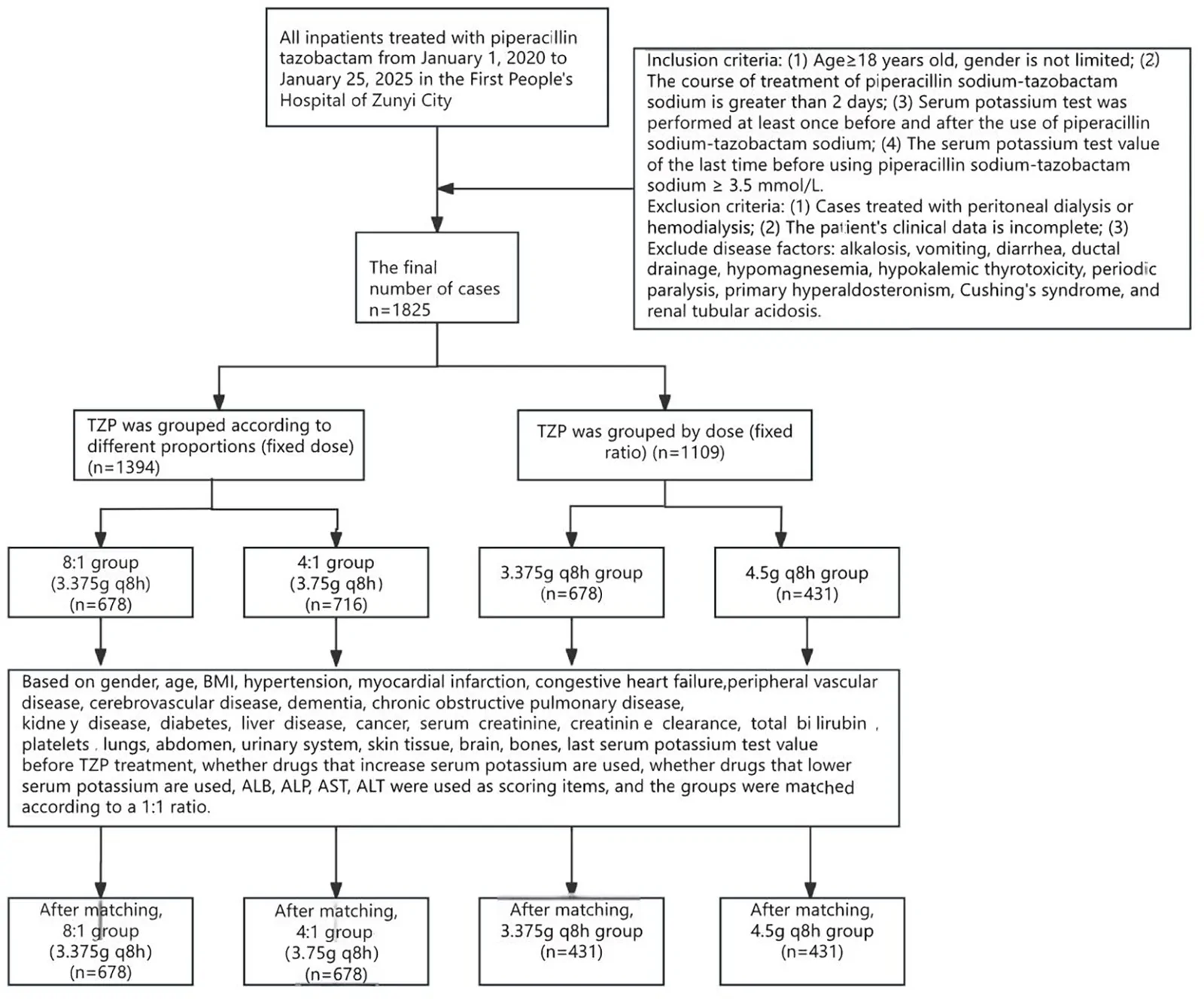
Flowchart of case screening and matching. PIMYN, use of medications that increase serum potassium; PLMYN, use of medications that decrease serum potassium; K_preTZP, last serum potassium value before TZP treatment; Ske, skeletal system infection; Cereb, cerebral infection; Cut, skin and soft tissue infection; Uro, urinary system infection; Abd, abdominal infection; Pul, pulmonary infection; PLT, platelet count; Tbil, total bilirubin; CrCl, creatinine clearance; Cr, serum creatinine; ALT, alanine aminotransferase; AST, aspartate aminotransferase; ALP, alkaline phosphatase; ALB, albumin; CA, cancer; LD, liver disease; DM, diabetes mellitus; KD, kidney disease; COPD, chronic obstructive pulmonary disease; DEM, dementia; CVD, cerebrovascular disease; PVD, peripheral vascular disease; CHF, congestive heart failure; MI, myocardial infarction; HTN, hypertension; BMI, body mass index; Age, age; Gender, sex.

**Figure 2:**
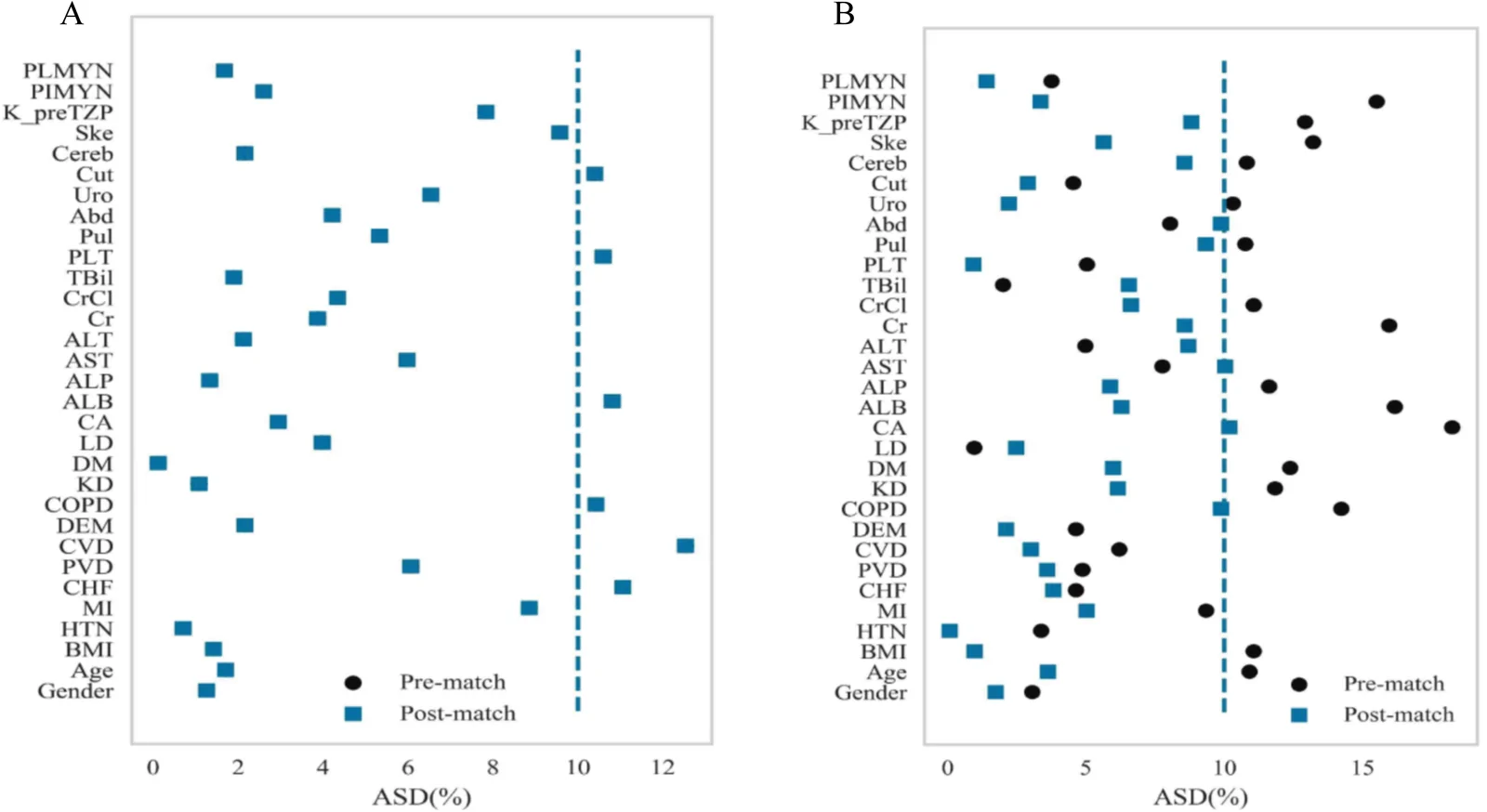
ASD scatter plots for different piperacillin/tazobactam ratio groups **(A)** and dosage groups **(B)**.

For the dosage comparison, 1,109 cases were included, comprising 678 cases in the TZP 3.375 g q8h group and 431 cases in the TZP 4.5 g q8h group. After 1:1 PSM, 862 cases remained for the final analysis, with 431 cases in each group. Before matching, the ASD values of several covariates exceeded 10%. After matching, the ASD values decreased substantially and were generally below 10%, indicating improved balance in the measured baseline characteristics, as shown in **Figure 2B**. Residual confounding by unmeasured clinical severity or treatment-selection factors remains possible.

### 3.2 Comparison of baseline characteristics before and after matching between the two TZP ratio groups

The results shown in **Table 3** indicated that, both before and after PSM, 31 variables, including sex and hypertension, showed no statistically significant differences between the two TZP ratio groups by chi-square test, with all *P* values > 0.05.

**Table 3:** Comparison of baseline characteristics before and after matching between patients receiving different TZP ratios.

Variable	Before matching			After matching		
	8:1group (n=678)	4:1group (n=716)	*P*-value	8:1group (n=678)	4:1group (n=678)	*P*-value
Male, n (%)	349 (51.475)	375 (52.374)	0.737	349 (51.475)	356 (52.507)	0.704
Age, mean (± SD)	71.680 ± 11.977	71.922 ± 11.817	0.705	71.680 ± 11.977	71.841 ± 11.835	0.804
BMI, mean (± SD)	21.754 ± 3.756	21.779 ± 3.400	0.897	21.754 ± 3.756	21.783 ± 3.396	0.882
ALB, mean (± SD)	34.862 ± 5.096	34.311 ± 5.395	0.054	34.862 ± 5.096	34.333 ± 5.403	0.066
ALP, mean (± SD)	101.232 ± 69.038	99.613 ± 49.678	0.619	101.232 ± 69.038	99.923 ± 49.956	0.692
AST, mean (± SD)	64.959 ± 504.823	44.979 ± 116.696	0.309	64.959 ± 504.823	45.595 ± 118.673	0.332
ALT, mean (± SD)	35.255 ± 173.989	33.439 ± 93.414	0.809	35.255 ± 173.989	34.080 ± 94.921	0.878
Cr, mean (± SD)	100.917 ± 103.491	96.773 ± 97.946	0.447	100.917 ± 103.491	96.263 ± 97.598	0.398
CrCl, mean (± SD)	66.865 ± 35.930	68.410 ± 36.087	0.429	66.865 ± 35.930	68.796 ± 36.442	0.33
TBil, mean (± SD)	16.306 ± 30.632	16.037 ± 10.908	0.828	16.306 ± 30.632	15.976 ± 10.831	0.793
PLT, mean (± SD)	216.317 ± 163.147	203.213 ± 94.619	0.071	216.317 ± 163.147	202.719 ± 94.640	0.064
K_preTZP, mean (± SD)	4.080 ± 0.450	4.107 ± 0.462	0.266	4.080 ± 0.450	4.108 ± 0.455	0.252
HTN, n (%)	322 (47.493)	334 (46.648)	0.752	322 (47.493)	317 (46.755)	0.752
MI, n (%)	53 (7.817)	72 (10.070)	0.141	53 (7.817)	68 (10.044)	0.151
CHF, n (%)	37 (5.457)	24 (3.352)	0.055	37 (5.457)	23 (3.392)	0.064
PVD, n (%)	61 (8.997)	75 (10.475)	0.353	61 (8.997)	72 (10.619)	0.315
DEM, n (%)	3 (0.442)	5 (0.698)	0.527	3 (0.442)	3 (0.442)	1.000
CVD, n (%)	142 (20.944)	176 (24.581)	0.106	142 (20.944)	171 (25.221)	0.062
COPD, n (%)	467 (68.879)	464 (64.804)	0.106	467 (68.879)	440 (64.897)	0.119
KD, n (%)	108 (15.929)	106 (14.804)	0.56	108 (15.929)	103 (15.192)	0.708
DM, n (%)	102 (15.044)	103 (14.385)	0.729	102 (15.044)	97 (14.307)	0.701
LD, n (%)	72 (10.619)	72 (10.056)	0.73	72 (10.619)	69 (10.177)	0.790
CA, n (%)	65 (9.587)	75 (10.475)	0.581	65 (9.587)	71 (10.472)	0.588
Pul, n (%)	676 (99.705)	713 (99.581)	0.699	676 (99.705)	675 (99.558)	0.654
Abd, n (%)	2 (0.295)	4 (0.559)	0.452	2 (0.295)	4 (0.590)	0.413
Uro, n (%)	78 (11.504)	67 (9.358)	0.189	78 (11.504)	65 (9.587)	0.25
Cut, n (%)	24 (3.540)	14 (1.955)	0.069	24 (3.540)	13 (1.917)	0.067
Cereb, n (%)	2 (0.295)	3 (0.419)	0.699	2 (0.295)	3 (0.442)	0.654
Ske, n (%)	10 (1.475)	4 (0.559)	0.086	10 (1.475)	4 (0.590)	0.107
PIMYN, n (%)	400 (58.997)	413 (57.682)	0.619	400 (58.997)	394 (58.112)	0.741
PLMYN, n(%)	390 (57.522)	414 (57.821)	0.91	390 (57.522)	392 (57.817)	0.912

PSM, propensity score matching; BMI, body mass index; ALB, albumin; ALP, alkaline phosphatase; AST, aspartate aminotransferase; ALT, alanine aminotransferase; Cr, serum creatinine; CrCl, creatinine clearance; TBil, total bilirubin; PLT, platelet count; K_preTZP, last serum potassium value before TZP treatment; HTN, hypertension; MI, myocardial infarction; CHF, congestive heart failure; PVD, peripheral vascular disease; DEM, dementia; CVD, cerebrovascular disease; COPD, chronic obstructive pulmonary disease; KD, kidney disease; DM, diabetes mellitus; LD, liver disease; CA, cancer; Pul, pulmonary infection; Abd, abdominal infection; Uro, urinary system infection; Cut, skin and soft tissue infection; Cereb, cerebral infection; Ske, skeletal system infection; PIMYN, use of medications that may increase serum potassium; PLMYN, use of medications that may decrease serum potassium.

In the matched cohort, the duration of TZP treatment was comparable between the two TZP ratio groups [median (IQR): 8.000 (6.000-10.900) days in the 4:1 group vs. 8.000 (6.000-10.000) days in the 8:1 group], and no statistically significant difference was observed. The detailed duration of TZP treatment, including values for the non-hypokalemia and hypokalemia subgroups, is presented in **Supplementary Table S1**. The follow-up window for each patient was defined from the index date (i.e., the date of TZP initiation) through the documented end of TZP treatment.

As shown in **Table 4**, after baseline characteristics were balanced by PSM, the incidence of hypokalemia was compared between the two TZP ratio groups under the condition of a fixed piperacillin dose. The incidence of hypokalemia was 33.186% in the TZP 8:1 ratio group (3.375 g q8h) and 33.481% in the TZP 4:1 ratio group (3.75 g q8h). The chi-square value was 0.013, and no significant difference in the incidence of hypokalemia was observed between the two groups, with no statistical significance (*P* > 0.05).

**Table 4:** Incidence of hypokalemia associated with different TZP ratios after PSM.

Group	Hypokalemia occurrence		Total	Hypokalemia incidence	Chi-square value	P-value
	1	0				
8:1 group	225	453	678	33.186%	0.013	0.908
4:1 group	227	451	678	33.481%		
Total	452	904	1356	33.333%		

0 indicates the number of cases without hypokalemia, while 1 represents the number of cases with hypokalemia.

### 3.3 Comparison of baseline characteristics before and after matching between the two TZP dosage groups

As shown in **Table 5**, before PSM, the two TZP dosage groups differed significantly in several baseline variables, including age, BMI, ALB, serum creatinine, myocardial infarction, chronic obstructive pulmonary disease, cancer, pulmonary infection, bone infection, and use of medications that increase serum potassium (P < 0.05). In contrast, sex, hypertension, congestive heart failure, ALP, AST, ALT, and other variables did not differ significantly between the two groups (P > 0.05). After matching, no statistically significant differences were observed between the two groups for any of the 31 baseline variables (P > 0.05).

**Table 5:** Comparison of baseline data before and after matching between two groups of patients receiving different doses of TZP.

Variable	Before matching			After matching		
	3.375g q8h group (n=678)	4.5g q8h group (n=431)	P-value	3.375g q8h group (n=431)	4.5g q8h group (n=431)	*P*-value
Male, n (%)	349 (51.475)	228 (52.900)	0.643	229 (53.132)	228 (52.900)	0.946
Age, Mean (± SD)	71.680 ± 11.977	69.673 ± 12.732	0.008	70.870 ± 12.735	69.673 ± 12.732	0.168
BMI, Mean (± SD)	21.754 ± 3.756	22.176 ± 3.218	0.047	22.127 ± 3.899	22.176 ± 3.218	0.841
ALB, Mean (± SD)	34.862 ± 5.096	35.884 ± 6.355	0.005	35.479 ± 5.041	35.884 ± 6.355	0.319
ALP, Mean (± SD)	101.232 ± 69.038	108.970 ± 97.762	0.145	105.119 ± 78.221	108.970 ± 97.762	0.542
AST, Mean (± SD)	64.959 ± 504.823	41.453 ± 100.209	0.363	81.604 ± 629.500	41.453 ± 100.209	0.213
ALT, Mean (± SD)	35.255 ± 173.989	30.517 ± 57.589	0.602	42.636 ± 216.085	30.517 ± 57.589	0.283
Cr, Mean (± SD)	100.917 ± 103.491	89.892 ± 78.730	0.049	93.459 ± 87.494	89.892 ± 78.730	0.537
CrCl, Mean (± SD)	66.865 ± 35.930	71.542 ± 59.713	0.11	69.635 ± 38.038	71.542 ± 59.713	0.581
TBil, Mean (± SD)	16.306 ± 30.632	15.852 ± 18.658	0.791	17.872 ± 37.937	15.852 ± 18.658	0.343
PLT, Mean (± SD)	216.317 ± 163.147	208.942 ± 103.898	0.421	208.788 ± 96.357	208.942 ± 103.898	0.982
K_preTZP, Mean (± SD)	4.080 ± 0.450	4.034 ± 0.439	0.093	4.059 ± 0.444	4.034 ± 0.439	0.405
HTN, n (%)	322 (47.493)	186 (43.155)	0.158	194 (45.012)	186 (43.155)	0.583
MI, n (%)	53 (7.817)	18 (4.176)	0.016	28 (6.497)	18 (4.176)	0.13
CHF, n (%)	37 (5.457)	33 (7.657)	0.142	24 (5.568)	33 (7.657)	0.217
PVD, n (%)	61 (8.997)	48 (11.137)	0.243	39 (9.049)	48 (11.137)	0.309
CVD, n (%)	142 (20.944)	79 (18.329)	0.288	85 (19.722)	79 (18.329)	0.603
DEM, n (%)	3 (0.442)	4 (0.928)	0.32	2 (0.464)	4 (0.928)	0.413
COPD, n (%)	467 (68.879)	269 (62.413)	0.026	291 (67.517)	269 (62.413)	0.116
KD, n (%)	108 (15.929)	82 (19.026)	0.182	73 (16.937)	82 (19.026)	0.425
DM, n (%)	102 (15.044)	79 (18.329)	0.149	74 (17.169)	79 (18.329)	0.656
LD, n (%)	72 (10.619)	52 (12.065)	0.457	52 (12.065)	52 (12.065)	1
CA, n (%)	65 (9.587)	60 (13.921)	0.026	50 (11.601)	60 (13.921)	0.307
Pul, n (%)	676 (99.705)	425 (98.608)	0.035	430 (99.768)	425 (98.608)	0.058
Abd, n (%)	2 (0.295)	1 (0.232)	0.844	2 (0.464)	1 (0.232)	0.563
Uro, n (%)	78 (11.504)	35 (8.121)	0.069	42 (9.745)	35 (8.121)	0.403
Cut, n (%)	24 (3.540)	11 (2.552)	0.359	14 (3.248)	11 (2.552)	0.543
Cereb, n (%)	2 (0.295)	5 (1.160)	0.076	2 (0.464)	5 (1.160)	0.255
Ske, n (%)	10 (1.475)	1 (0.232)	0.042	3 (0.696)	1 (0.232)	0.316
PIMYN, n (%)	400 (58.997)	282 (65.429)	0.032	281 (65.197)	282 (65.429)	0.943
PLMYN, n (%)	390 (57.522)	235 (54.524)	0.326	234 (54.292)	235 (54.524)	0.945

PSM, propensity score matching; BMI, body mass index; ALB, albumin; ALP, alkaline phosphatase; AST, aspartate aminotransferase; ALT, alanine aminotransferase; Cr, serum creatinine; CrCl, creatinine clearance; TBil, total bilirubin; PLT, platelet count; K_preTZP, last serum potassium value before TZP treatment; HTN, hypertension; MI, myocardial infarction; CHF, congestive heart failure; PVD, peripheral vascular disease; DEM, dementia; CVD, cerebrovascular disease; COPD, chronic obstructive pulmonary disease; KD, kidney disease; DM, diabetes mellitus; LD, liver disease; CA, cancer; Pul, pulmonary infection; Abd, abdominal infection; Uro, urinary system infection; Cut, skin and soft tissue infection; Cereb, cerebral infection; Ske, skeletal system infection; PIMYN, use of medications that may increase serum potassium; PLMYN, use of medications that may decrease serum potassium.

In the matched cohort, the duration of TZP treatment differed significantly between the two TZP dosage groups, being significantly shorter in the 4.5 g q8h group than in the 3.375 g q8h group [median (IQR): 6.9 (5.0-9.0) days vs. 7.8 (5.8-10.0) days; *P* < 0.05]. The detailed duration of TZP treatment, including values for the non-hypokalemia and hypokalemia subgroups, is presented in **Supplementary Table S1**. The follow-up window for each patient was defined from the index date (i.e., the date of TZP initiation) through the documented end of TZP treatment.

As shown in **Table 6**, after PSM was used to balance the measured baseline characteristics of the two groups, the chi-square test was applied to compare the incidence of hypokalemia between different TZP dosage groups under a fixed component ratio of 8:1. The incidence of hypokalemia was 33.643% in patients receiving TZP 3.375 g q8h and 40.139% in patients receiving TZP 4.5 g q8h. The chi-square value was 3.907, and the difference was statistically significant (*P* = 0.048). Notably, despite a significantly shorter duration of TZP treatment, patients in the 4.5 g q8h group exhibited a higher incidence of hypokalemia than those in the 3.375 g q8h group, further supporting a dose-related effect of TZP on hypokalemia. Given the observational design, the present findings reflect an adjusted association between TZP dosage and hypokalemia after accounting for measured covariates; however, residual confounding by unmeasured factors precludes any causal interpretation of a dose-response effect.

**Table 6:** Incidence of hypokalemia associated with different TZP doses after PSM.

Group	Hypokalemia occurrence		Total	Hypokalemia incidence	Chi-square value	*P*-value
	1	0				
4.5g q8h group	173	258	431	40.139%	3.907	0.048
3.375g q8h group	145	286	431	33.643%		
Total	318	544	862	36.891%		

0 indicates the number of cases without hypokalemia, while 1 represents the number of cases with hypokalemia.

### 3.4 Hypokalemia induced by penicillin-class antibiotics

As shown in **Figure 3**, among the penicillin-class antibiotics included in the analysis, 36 hypokalemia-related reports were identified for TZP, 20 for amoxicillin/clavulanic acid, 46 for amoxicillin, 18 for ampicillin, and 5 for oxacillin, whereas no related reports were identified for penicillin G. TZP and ampicillin met the prespecified disproportionality criteria for positive hypokalemia reporting signals. The ROR (95% CI) and PRR (95% CI) for TZP were 3.809 (2.744-5.287) and 3.786 (2.733-5.245), respectively, with χ² = 71.205. For ampicillin, the ROR (95% CI) and PRR (95% CI) were 5.326 (3.348-8.473) and 5.287 (3.337-8.375), respectively, with χ² = 58.414. Oxacillin had high point estimates but only five reports, limiting signal stability. Amoxicillin/clavulanic acid did not meet the full MHRA signal criteria consistently, and the ROR and PRR values for amoxicillin were approximately 0.98 with 95% CIs including 1. These results indicate that TZP and ampicillin exhibited disproportional reporting of hypokalemia relative to other database reports, generating a signal of a potential association that merits further investigation; they do not establish the comparative frequency or severity of hypokalemia across antibiotics.

**Figure 3:**
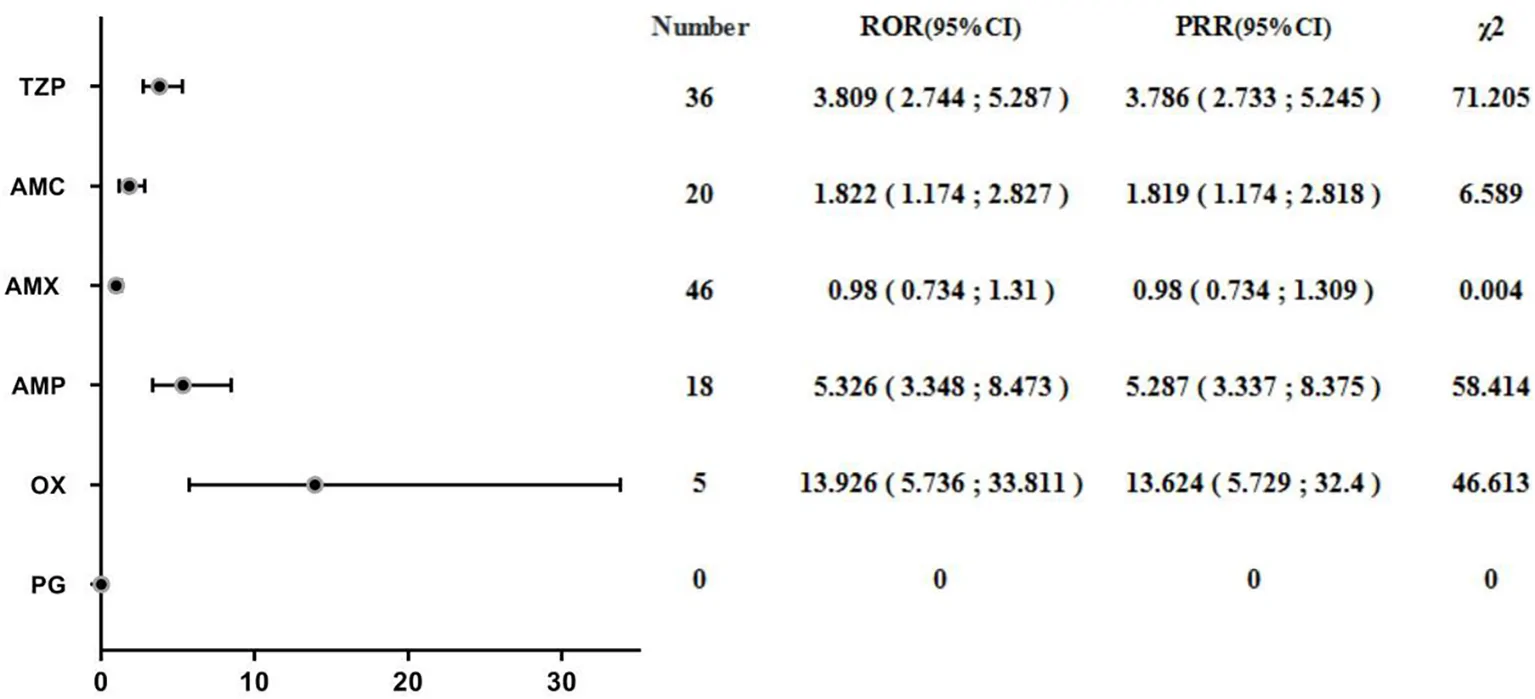
Forest plot of hypokalemia reporting disproportionality for selected penicillin-class antibiotics. TZP, piperacillin/tazobactam; AMC, amoxicillin/clavulanic acid; AMX, amoxicillin; AMP, ampicillin; OX, oxacillin; PG, penicillin G; ROR, reporting odds ratio; PRR, proportional reporting ratio; CI, confidence interval.

## 4 Discussion

Previous clinical studies (Wang et al., 2026; Seo and Kim, 2022; Cao et al., 2023) and pharmacovigilance analyses (Seo and Kim, 2023) have raised concern regarding hypokalemia during TZP therapy. The present study combined a single-center retrospective cohort with a separate FAERS disproportionality analysis. In the clinical cohort, PSM was used to compare different TZP component ratios and dosage regimens after balancing measured covariates. The ratio comparison showed no detectable difference in hypokalemia incidence, whereas the higher-dose regimen was associated with a higher observed incidence after matching. In the pharmacovigilance analysis, TZP and ampicillin showed positive disproportionality signals for hypokalemia. Importantly, the cohort and FAERS components answer different questions: the cohort estimates within-TZP differences in observed incidence, whereas FAERS detects disproportionate reporting and cannot establish incidence or causal risk.

In the ratio-group analysis, most baseline indicators showed no significant deviation before or after matching, suggesting no strong association between the severity of hypokalemia and these indicators. After PSM, when the piperacillin dose was fixed, the incidence of hypokalemia was 33.186% in the 8:1 ratio group and 33.481% in the 4:1 ratio group, with a chi-square value of 0.013. The difference between the two groups was not statistically significant (*P* > 0.05). These findings indicate that, under the same piperacillin dose, the component ratio itself is not a key determinant of the risk of TZP-induced hypokalemia. The physiological and pharmacokinetic basis for this observation deserves further consideration. First, as a combination preparation, TZP contains both tazobactam and piperacillin, which do not share completely identical metabolic profiles *in vivo*. Although the proportion of tazobactam was increased, the incidence of hypokalemia did not change significantly. This finding suggests that hypokalemia is unlikely to be driven mainly by the direct effect of tazobactam, but is more likely related to the chemical properties of piperacillin itself. Second, the influence of component-ratio differences on blood drug concentrations may be limited. When the piperacillin exposure received by patients in the two groups is generally comparable, changes in the component ratio are unlikely to produce a substantial difference in the concentration of piperacillin to which renal tubular epithelial cells are exposed. Therefore, even when the proportion of tazobactam increases, its competitive inhibitory effect on renal tubular organic anion transporters (OATs) does not appear to markedly alter the degree of piperacillin accumulation in renal tubules (Wang et al., 2026). This further supports the view that piperacillin itself, rather than the proportion of tazobactam, is the key factor involved in electrolyte disturbances. In addition, Hu et al. (2024) reported no statistically significant difference in adverse reactions between the TZP 8:1 group and the TZP 4:1 group (*P* = 0.605), and the main adverse reactions of TZP included elevated liver enzymes, increased creatinine, and electrolyte disturbances. The present study focused specifically on hypokalemia as one type of electrolyte disturbance, and the findings are consistent with those reported by Hu et al. (2024).

In the dosage-group analysis, most baseline variables were well balanced after PSM. We compared TZP 3.375 g q8h and 4.5 g q8h under a fixed component ratio of 8:1 and found that the incidence of hypokalemia was significantly higher in the higher-dose group, namely the 4.5 g q8h group (*P* < 0.05). Specifically, the incidence was 33.643% in the 3.375 g q8h group and 40.139% in the 4.5 g q8h group, with a chi-square value of 3.907, indicating a statistically significant difference between the two groups (*P* < 0.05). The retrospective study by Seo and Kim (2022) clearly showed that a higher daily dose of TZP was a significant independent risk factor for TZP-associated hypokalemia. Although that study did not directly compare 3.375 g q8h with 4.5 g q8h, the dose-response relationship it reported strongly supports the view that a higher dose is associated with a positive disproportionality signal. This phenomenon may be explained by an increase in blood drug concentration beyond a certain threshold, which may lead to saturation of renal tubular organic anion transporters (OATs), intratubular drug accumulation, and accelerated potassium excretion. Taken together with the findings of Wang et al., (2026); Seo and Kim (2022), this dose-response relationship supports the conclusion that “high dose and long treatment duration” are independent risk factors for hypokalemia. In clinical practice, priority should therefore be given to controlling the cumulative dose and treatment duration to reduce the risk of drug-associated hypokalemia.

Several studies (Jansen et al., 2024; Seo and Kim, 2023; Isaac et al., 2025) have confirmed that penicillin-class antibiotics can induce clinically significant hypokalemia. This study included six penicillin-class antibiotics, including TZP, amoxicillin/clavulanic acid, and amoxicillin. Except for penicillin G, all of these drugs had adverse event reports related to hypokalemia. Notably, TZP and ampicillin showed a significantly higher risk of hypokalemia than other drugs in the same class, and the number of reported cases was also higher than that for most other agents. These two drugs have similar molecular configurations, suggesting that this adverse reaction may be closely related to their specific chemical structures. The core mechanism of β-lactam antibiotic-induced hypokalemia mainly involves renal tubular transport. Taking TZP as an example, it contains a free carboxyl group and a β-lactam nucleus, which are readily recognized and taken up by organic anion transporters, such as OAT1 and OAT3; the polar groups in its side chain may further enhance this process (Alosaimy et al., 2024; Wen et al., 2018; Mazerkina et al., 2020). The α-amino group of ampicillin makes it prone to forming a zwitterion at physiological pH, and in alkaline urine it tends to exist in an anionic form, which also increases OAT-mediated accumulation (Zhang et al., 2023; Liu et al., 2026; Dong et al., 2025), ultimately leading to high intratubular drug concentrations. High-concentration drug accumulation may induce hypokalemia through several mechanisms: (1) hydrophobic β-lactams, such as piperacillin, have affinity for OAT3, resulting in increased uptake and nephrotoxicity, which impairs renal tubular function and affects ion reabsorption, indirectly causing potassium loss (Alosaimy et al., 2024); (2) high drug concentrations may inhibit Na^+^-K^+^-ATPase activity and interfere with the function of ROMK channels, which are key potassium-secreting channels, and dysfunction of these channels can increase potassium excretion (Jansen et al., 2024); and (3) some β-lactam antibiotics may induce mitochondrial oxidative stress, damage cells, and downregulate the expression of basolateral potassium channels, such as Kir4.1/Kir5.1, thereby further inhibiting potassium reabsorption and collectively aggravating hypokalemia (Yang et al., 2023; Su and Wang, 2016; Zhang et al., 2021). Comparative analysis with other penicillin-class antibiotics showed that, in amoxicillin/clavulanic acid, clavulanic acid has high affinity for OATs, but its short half-life limits renal tubular accumulation, resulting in only a weak association with hypokalemia (Chen and Wu, 2024). Amoxicillin lacks an α-amino group, and its OAT transport efficiency is lower than that of ampicillin; accordingly, it did not show a significant association (Tashiro et al., 2025). Oxacillin is mainly metabolized by hepatic CYP enzymes, resulting in low renal tubular exposure (Konçe and Demiralay, 2023). In this study, oxacillin showed a relatively high reporting ratio for hypokalemia, but the small number of cases limited the statistical stability of this result. Penicillin G, as the earliest penicillin-class antibiotic, is also excreted by the kidney (Spencer, 2019); however, its short half-life and limited renal tubular accumulation may explain its relatively low incidence of hypokalemia. The above structure-activity relationships are highly consistent with the ROR/PRR differences observed in this study. Specifically, TZP and ampicillin may exert a more pronounced effect on renal tubular ion transport because of their specific chemical structures, particularly the α-amino group and polar side-chain groups, thereby leading to a higher risk of hypokalemia.

This study has several limitations. First, PSM can balance only measured covariates and cannot eliminate residual confounding. This is particularly important for the dosage comparison because higher-dose TZP may be preferentially prescribed to patients with more severe infection, suspected Pseudomonas infection, sepsis, or rapidly changing physiology. Standardized disease-severity scores, microbiological indications, and clinician treatment-selection factors were not included in the matching model, so confounding by indication remains possible. Second, the concomitant medication variables PIMYN and PLMYN grouped heterogeneous drug classes into broad binary categories. Potassium-containing intravenous fluids were not separately captured as a time-varying exposure; therapeutic potassium administration could have prevented some serum potassium values from falling below the predefined threshold and therefore may have led to under-ascertainment of hypokalemia. Third, the local cohort included only TZP-treated patients and therefore provides internal comparisons of component ratio and dosage; it cannot establish whether TZP has a uniquely higher clinical incidence of hypokalemia than cephalosporins or other penicillins. Fourth, although TZP treatment duration was extracted, length of hospital stay was not included as a separate analytic population characteristic, limiting description of the overall observation period. Fifth, FAERS is a spontaneous reporting database subject to underreporting, stimulated reporting, notoriety bias, duplicate or incomplete reports, missing clinical covariates, variable data quality, and lack of a reliable exposed population denominator. Consequently, ROR and PRR quantify reporting disproportionality rather than incidence or relative clinical risk, and they cannot establish causality or fully control confounding. Finally, this was a single-center retrospective study, and the more common use of the 8:1 regimen produced unequal group sizes and loss of some observations after matching. Multicenter studies with active comparators, detailed time-varying treatment data, and prospective collection of disease-severity measures are needed to validate these findings.

## 5 Conclusion

Based on large-scale real-world data, this study used PSM to strictly control for confounding factors and systematically examined the effects of different TZP component ratios and dosages on the risk of hypokalemia. The results showed that component-ratio differences, namely 8:1 versus 4:1, were not a key factor affecting the incidence of hypokalemia, and the risks were comparable between the two groups under the same dosage condition. However, increased dosage was significantly and positively associated with the incidence of hypokalemia. Specifically, the risk in the 4.5 g q8h group was significantly higher than that in the 3.375 g q8h group, which is highly consistent with previous findings that “high dose and long treatment duration” are independent risk factors. Among penicillin-class antibiotics, TZP and ampicillin were positive disproportionality signal, which may be related to their specific *β*-lactam structures. These structures may interfere with renal tubular organic anion transporters (OATs) and key potassium channels, such as ROMK, thereby impairing potassium reabsorption or increasing potassium excretion. In summary, clinical medication use should focus on the cumulative dose and treatment duration of TZP rather than its component ratio, in order to reduce the risk of drug-associated hypokalemia.

## Data Availability

The original contributions presented in the study are included in the article/**Supplementary Material**. Further inquiries can be directed to the corresponding author.
